# Long Noncoding RNA *HOTAIR* as an Independent Prognostic Marker in Cancer: A Meta-Analysis

**DOI:** 10.1371/journal.pone.0105538

**Published:** 2014-08-26

**Authors:** Shenghong Zhang, Shuling Chen, Guang Yang, Fang Gu, Minrui Li, Bihui Zhong, Jifan Hu, Andrew Hoffman, Minhu Chen

**Affiliations:** 1 Division of Gastroenterology, The First Affiliated Hospital, Sun Yat-sen University, Guangzhou, P.R. China; 2 Division of Reproductive Medicine, The First Affiliated Hospital, Sun Yat-sen University, Guangzhou, P.R. China; 3 VA Palo Alto Health Care System and Stanford University Medical School, Palo Alto, California, United States of America; Central South University, China

## Abstract

**Background:**

*HOTAIR*, a newly discovered long intergenic noncoding RNA (lincRNA), has been reported to be aberrantly expressed in many types of cancers. This meta-analysis summarizes its potential role as a biomarker in malignancy.

**Methods:**

A quantitative meta-analysis was performed through a systematic search in Pubmed, Medline and Web of Science for eligible papers on the prognostic impact of *HOTAIR* in cancer from inception to Feb. 28, 2014. Pooled hazard ratios (HRs) with 95% confidence interval (95% CI) were calculated to summarize the effect.

**Results:**

Nineteen studies were included in the study, with a total of 2033 patients. A significant association was observed between high *HOTAIR* expression and poor overall survival (OS) in patients with cancer (pooled HR 2.22, 95% CI: 1.68–2.93). Place of residence (Asian or Western countries), type of cancer (digestive or non-digestive disease), sample size (more or less than 100), and paper quality (score more or less than 85%) did not alter the significant predictive value of *HOTAIR* in OS from various kinds of cancer but preoperative status did. By combining HRs from Cox multivariate analyses, we found that *HOTAIR* expression was an independent prognostic factor for cancer patients (pooled HR 2.26, 95% CI: 1.62–3.15). Subgroup analysis showed that *HOTAIR* abundance was an independent prognostic factor for cancer metastasis (HR 3.90, 95% CI: 2.25–6.74). For esophageal carcinoma, high *HOTAIR* expression was significantly associated with TNM stage (III/IV vs. I/II: OR 6.90, 95% CI: 2.81–16.9) without heterogeneity. In gastric cancer, *HOTAIR* expression was found to be significantly associated with lymph node metastases (present vs. absent: OR 4.47, 95% CI: 1.88–10.63) and vessel invasion (positive vs. negative: OR 2.88, 95% CI: 1.38–6.04) without obvious heterogeneity.

**Conclusions:**

*HOTAIR* abundance may serve as a novel predictive factor for poor prognosis in different types of cancers in both Asian and Western countries.

## Introduction

GLOBOCAN 2012 reports that an estimated 14.1 million new cancer cases and 8.2 million cancer deaths occurred in 2012, and most of them occurred in less developed countries [1]. Cancer has now become a major cause of morbidity and mortality in most regions worldwide [2]. The 5-year survival rate remains low in many types of cancers, and numerous investigators are searching for biomarkers that may help with diagnosis or prognosis of cancer [3].

Recently, genome-wide transcriptome studies have confirmed that there are a large number of long intergenic noncoding RNAs (lincRNAs), which in the past had been dismissed as simply transcriptional “noise” [4]. LincRNAs are non-protein coding RNA molecules greater than 200 nucleotides in length. Diverse biological functions, including cell differentiation, development and many disease processes, have been attributed to lincRNAs. *HOTAIR* is a lincRNA that is crucial for cell growth and viability [5], [6]. It is transcribed from the antisense strand of the *HOXC* gene on chromosome 12q13.13 [5]. *HOTAIR* has been implicated in cancer invasion and metastasis through its role in chromatin remodeling. By targeting polycomb repressive complex 2 (PRC2) and LSD1 complexes to chromatin for coupled histone methylation and demethylation processes, *HOTAIR* silences various target genes, including the HOXD cluster [5].

*HOTAIR* is aberrantly expressed in a variety of human cancers, including breast cancer, colorectal cancer, laryngeal squamous cell carcinoma, and liver cancer [6]–[9]. It has been suggested that *HOTAIR* expression may play a useful prognostic role in some tumors. However, most studies examining the implications of *HOTAIR* expression are limited by small sample size. Therefore, we conducted a systematic review and quantitative meta-analysis to clarify the prognostic value of *HOTAIR* expression in human cancers.

## Materials and Methods

### Study strategy

The present review was performed in accordance with the standard guidelines for meta-analyses and systematic reviews of tumor marker prognostic studies [10], [11]. To obtain relevant articles for this review, two authors (SH Zhang and SL Chen) independently used the following research tools: Medline, Pubmed, and Web of Science to identify all relevant articles about *HOTAIR* as a prognostic factor for survival of patients with any cancer. The literature search ended on Feb 28, 2014. The search strategy used both MeSH terms and free-text words to increase the sensitivity of the search. The following search terms were used: “*HOTAIR*”, “long intergenic noncoding RNA”, “lincRNA”, “lncRNA”, “noncoding RNA”, “cancer”, “carcinoma”, “neoplasm”, “prognosis”, “prognostic”, “outcome”, “mortality”, “survival”, and “recurrence”.

### Study selection

The same two investigators independently assessed all the eligible studies and extracted the data. Studies were considered eligible if they met the following criteria: any type of human cancer was studied; *HOTAIR* expression was determined in human tissue using quantitative PCR or microarray expression analysis; the relationship between *HOTAIR* expression and survival was examined; sufficient data was provided to estimate hazard ratios (HRs) for survival rates and their 95% confidence intervals. If data subsets were published in more than one article, only the most recent one was included. Citations were limited to those published in the English language. Animal studies [10] and single case reports were excluded [11]. If the data could not be extracted or calculated from the original article, the study was excluded. Disagreements were resolved through discussion with a third investigator (G Yang).

### Data extraction

The two investigators (SH Zhang and SL Chen) extracted data independently and reached a consensus on all items. For each study, the following characteristics of the individual research articles were collected: author, journal name, year of publication, country of the population enrolled, ethnicity, number of patients, study design, follow-up, overall survival (OS),methods, cut-off values, treatment data, disease-free survival (DFS), metastasis-free survival (MFS), and recurrence-free survival (RFS).

### Quality assessment of primary studies

Quality assessment was performed independently by three investigators (SH Zhang, SL Chen, and MH Chen). All eligible studies were scored as previously reported [12], [13]. The final scores are expressed as percentages, with a higher percentage denoting better methodological quality.

### Statistical analysis

We extracted HRs according to the following three methods [14]. The first and most accurate method was to obtain the reported HRs directly from the publication, or to estimate the HRs from O-E statistic and variance. If that was not possible, we calculated the HRs from the published data including the number of patients at risk in each group, the number of events and the log-rank statistic or its *p* value. However, there were still some HRs that could not be retrieved using the above methods, as they were presented in the form of Kaplan-Meier Curves. Therefore, with the assumption of a constant rate of the censored patients during follow-up, we reconstructed the HR estimate by extracting several survival rates at specified times from the survival curves. Since the approximation of the survival curves introduces error, we attempted to minimize this error by using the Engauge Digitizer version 2.11 to obtain the necessary points. We inputted the extracted survival rates at specified times into the spreadsheet developed by Tierney JF et al and estimated censoring using the minimum and maximum follow-up [14]. Then an approximated curve was produced; we compared it with published curves to confirm the accuracy of our data extraction and to assist in data adjustment [14]. If needed, we sought original data directly from the authors of the relevant studies.

Pooled hazard ratios or odds ratios (HRs or ORs) and their associated 95% confidence intervals (CI) were estimated using a fixed-effect model (Mantel-Haenszel), while the random effects model was performed when significant heterogeneity was present [15]. For each study, HR was estimated as previously reported [16]. The individual HR estimates were pooled into a summary HR using published methods [17]. Statistical heterogeneity among studies was assessed by using the I^2^ statistic, with significant heterogeneity defined as an I^2^>50% [18]. Subgroup analysis and meta regression by factor of region, sample size, type of cancer and paper quality score were both performed to determine if the number of included studies was sufficient. Univariate meta-regression was conducted to explore the potential heterogeneity in the analysis of the association between *HOTAIR* and survival. Furthermore, factors identified as significant by univariate analysis were further analyzed with multivariate meta-regression if necessary. We also conducted sensitivity analyses to test the effect of each study on the overall pooled results. The presence of publication bias was evaluated by using funnel plots, Begg's test and Egger's test [16]. Statistical analysis was performed using Stata software statistical software version 12.0 (Stata, College Station, TX). A *p* value of less than 0.05 was considered statistically significant.

## Results

### Included studies and characteristics

**Figure 1:**
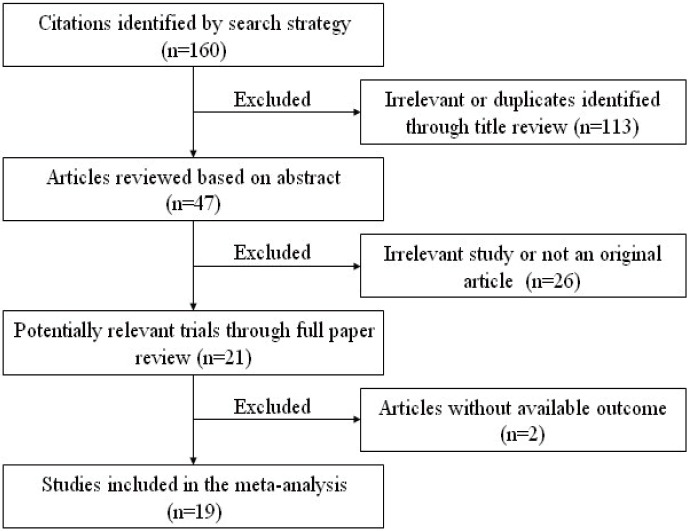
The flow diagram of the meta analysis.

As shown in the flow diagram (**Figure 1**), our search terms revealed 160 articles. After the titles and abstracts were reviewed, 113 irrelevant or duplicate articles were excluded. After a more careful inspection of the abstracts, a total of 21 articles were reviewed in detail [6]–[9], [19]–[35]. Two papers were excluded because of insufficient data to estimate HR for further analysis [34], [35]. As a result, 19 published articles were included in the current meta-analysis [6]–[9], [19]–[33]. Among these 19 studies, a total of 2033 patients were included, with a maximum sample size of 292 and a minimum sample size of 39 patients (Mean 107.0). Nine studies enrolled more than 100 participants. The accrual period of these studies ranged from 2010 to 2014. The studies were published by groups throughout the world: 11 from China, 4 from Japan, 3 from the United States and 1 from Denmark. A total of 12 different types of cancer were evaluated in studies in this meta-analysis, with the greatest number being digestive system malignancies (4 esophageal carcinoma, 2 gastric cancer, 2 hepatocellular cancer, 1 colorectal cancer, 1 pancreatic cancer and 1 gastrointestinal stromal tumor); other types of cancer were also included (3 breast cancer, 2 non-small cell lung cancer, 1 nasopharyngeal carcinoma, 1 laryngeal carcinoma, 1 mesenchymal glioma and 1 endometrial carcinoma). Treatment information was not available in 4 studies and of the remaining researches, the participants in two received preoperative treatment.

**Table 1** summarizes the main characteristics of the included studies. A total of 21 HRs were analyzed. HRs from two studies were calculated by using one of the three methods noted in the Materials and Methods section. HRs could be obtained directly in seventeen studies, and HRs were approximated in two studies by using the total number of events and its *p*-value. We extrapolated the remaining HRs from two studies using graphical representations of the survival distributions.

**Table 1:** Characteristics of studies included in the meta-analysis.

Study	Year	Region	Tumor type	Sample size (n)	Clinical stage of tumor	Cut-off value	Elevated HOTAIR (%)	Preoperative treatment	Outcome measures	Survival analysis	Method*	Quality score (%)
Chen et al.	2013	China	Esophageal carcinoma	78	0-IV	26.6	34.6	No	OS	Multivariate analysis	1	85.0
Li et al.	2013	China	Esophageal carcinoma	100	I-IV	125-fold compared with NEECs	30	No	OS	Multivariate analysis	1	84.4
Ge et al.	2013	China	Esophageal carcinoma	137	N/A	N/A	65.7	No	OS, MFS	Multivariate analysis	1	78.1
Lv et al.	2013	China	Esophageal carcinoma	93	I-IV	an SI score of 6	52.7	No	OS	Multivariate analysis	1	72.5
Yang et al.	2011	China	Hepatocellular carcinoma	60	Within or beyond Milan criteria	N/A	53.3	NA	RFS	Multivariate analysis	1	85.0
Xu et al.	2013	China	Gastric cancer	83	I-IV	N/A	67.5	Yes	OS	Univariate and multivariate analysis	1	87.5
Endo et al.	2013	Japan	Gastric cancer	68	I-IV	HOTAIR/GAPDH of 1.0	63.2	No	OS	No	3	73.1
Gupta et al.	2010	USA	Breast cancer	132	I-IV	125-fold compared with normal tissue	33.3	NA	OS, MFS	Multivariate analysis	1	92.5
Lu et al.	2012	USA	Breast cancer	292	I-IV	10.01 EI (median level)	33.2	Yes	OS, DFS	Univariate and multivariate analysis	1	87.5
Sørensen et al.	2013	Denmark	Breast cancer	164	NA	Density plot of gene expression of 0.6	48.2	No	MFS	Multivariate analysis	1	90.0
Kogo et al.	2011	Japan	Colorectal cancer	100	Dukes' stage A,B,C,D	HOTAIR/GAPDH of 0.273	20.0	No	OS	Univariate and multivariate analysis	1	90.0
Kim et al.	2013	USA	Pancreatic cancer	102	I-IV	15% of HOTAIR expression	13.7	NA	OS	Multivariate analysis	1	81.3
Niinuma et al.	2012	Japan	Gastrointestinal stromal tumor	39	Very low, low, intermediate, high	HOTAIR/GAPDH of 0.0002	28.2	NA	OS	Univariate and multivariate analysis	1	85.0
Nakagawa et al.	2013	Japan	Non-small cell lung cancer	77	I-IV	2-fold compared with normal tissue	22.0	No	DFI	No	1	71.9
Liu et al.	2013	China	Non-small cell lung cancer	42	I-IV	HOTAIR/GAPDH of 8.57	50.0	No	OS	No	3	67.5
Li et al.	2013	China	Laryngeal carcinoma	72	I-IV	N/A	45.8	No	OS	Multivariate analysis	1	81.9
Nie etal.	2013	China	Naosopharyngeal carcinoma	160	I-IV	an SI score of 6	56.9	No	OS, DFS, LRFS,DMFS	Univariate and multivariate analysis	1,2,2	85.0
Zhang et al.	2013	China	Mesenchymal glioma	89	high grade/low grade	N/A	49.4	NA	OS	Univariate and multivariate analysis	1	87.5
He et al.	2013	China	Endometrial carcinoma	145	I-IV	an SI score of 6	42.8	No	OS	No	1	75.0

SI (staining index score): staining intensity x proportion of positively stained cells. OS: overall survival. DFS: disease free survival. MFS: metastasis free survival. LRFS: local recurrence free survival. DMFS: distant metastasis free survival.

EI: expression index, it was calculated from the formula 1,000 9 2(-DCt), where DCt = Ct (HOTAIR) - Ct (GAPDH). NA: not available.

*1 denoted as obtaining HRs directly from publications; 2 denoted as calculating HRs from the total number of events and its *p*-value; 3 denoted as extracting HRs from Kaplan-Meier curves.

All of the studies were comprised of a high *HOTAIR* expression arm and a low *HOTAIR* expression arm. The average percentage of tumors with increased *HOTAIR* expression was 42.0%, with a maximum of 67.5% in gastric cancer and a minimum of 13.7% in pancreatic cancer. OS, DFS, RFS and MFS were estimated as survival outcome measures in 84.2% (16/19), 5.26% (1/19), 15.8% (3/19) and 21.1% (4/19) of the studies, respectively. Multivariable analyses were performed in 84.2% (16/19) of studies and *HOTAIR* expression was found to be an independent prognostic factor for OS in 91.7% (11/12) of studies, for recurrence in 1 of 2 of studies, and for metastasis in 2 of 2 of studies.

### Association between *HOTAIR* and survival in twelve types of cancers

**Figure 2:**
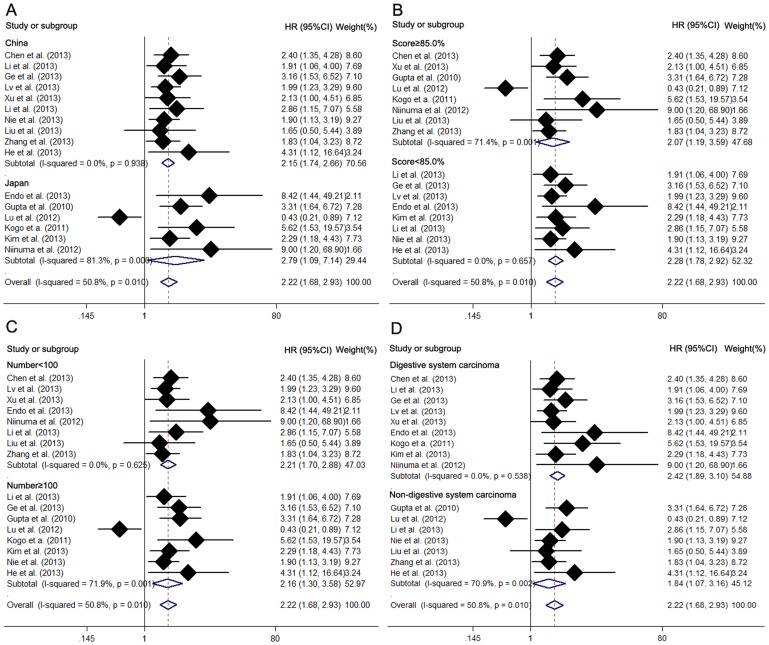
Meta analysis of the pooled HRs of OS of different types of cancer with increased *HOTAIR* expression. (A) Subgroup analysis of HRs of OS by factor of region. (B) Subgroup analysis of HRs of OS by factor of score. (C) Subgroup analysis of HRs of OS by factor of sample size. (D) Subgroup analysis of HRs of OS by factor of type of cancer.

Sixteen studies reported the overall survival (OS) of twelve types of cancer based on different *HOTAIR* expression levels in a total of 1732 patients. A significant association was observed between *HOTAIR* and OS in cancer patients (pooled HR 2.22, 95% CI: 1.68–2.93) (Figure 2). Significant heterogeneity existed between studies (χ^2^ = 30.47, df = 15, *p* = 0.010; I^2^ = 50.8%).

Due to the presence of heterogeneity, subgroups were analyzed based on the region, sample size, type of cancer, preoperative treatment and paper quality (**Table 2**) (**Figure 2**). We detected a significant association between *HOTAIR* and OS of cancer patients in both Asian (HR 2.15, 95%CI: 1.74–2.66) and western countries (HR 2.79, 95%CI: 1.09–7.14). *HOTAIR* was found to be significantly associated with OS of patients with digestive system malignancies (HR 2.42, 95%CI: 1.89–3.10) and with OS of patients with non-digestive system malignancies (HR 1.84, 95%CI: 1.07–3.16). The association between *HOTAIR* and OS of patients was present in studies with more than 100 or fewer than 100 subjects. After excluding the four studies without available treatment information, *HOTAIR* was found to be significantly associated with OS in patients without preoperative treatment (HR 2.34, 95% CI: 1.86–2.96) but not in those who received preoperative treatment (HR 0.953, 95% CI: 0.199–4.57). Paper quality did not change the result of the estimated HR (HR 2.07, 95% CI: 1.19–3.59; HR 2.28, 95%CI: 1.78–2.92 respectively), but in those studies with a paper quality score of less than 85.0, there was more heterogeneity across studies than in the subgroup with higher quality. Significant heterogeneity existed across studies in the subgroup of western countries, in the subgroup of patient number more than 100 and in the subgroup of patients with non-digestive system malignancy but there was not significant heterogeneity in the subgroups of Asian countries, patient number fewer than 100 and patients with digestive system malignancy. Thus, the region, type of cancer and sample size did not alter the significant predictive value of *HOTAIR* in OS of various kinds of cancer.

**Table 2:** Results of subgroup analysis of pooled hazard ratios of overall survival of different types of cancer with increased *HOTAIR* expression.

Subgroup analysis	No. of studies	No. of patients	Pooled HR(95%CI)		Meta regression (*p*-value)	Heterogeneity	
			Fixed	Random		I^2^	*p*-value
**Region**							
Asian countries	13	1206	2.15[1.74–2.66]	2.15[1.74–2.66]	0.98	0.00%	0.938
Western countries	3	526	1.96[1.36–2.83]	2.79[1.09–7.14]		81.3%	0.000
**Sample size**							
<100	8	564	2.21[1.70–2.88]	2.21[1.70–2.88]	0.145	0.00%	0.625
≥100	8	1168	2.00[1.55–2.59]	2.16[1.30–3.58]		71.9%	0.001
**Type of cancer**							
Digestive system carcinoma	9	800	2.42[1.89–3.10]	2.42[1.89–3.10]	0.071	0.00%	0.538
Non-digestive system carcinoma	7	932	1.76[1.34–2.32]	1.84[1.07–3.16]		70.9%	0.002
**Preoperative treatment**	12	1611	2.01[1.62–2.48]	2.13[1.50–3.04]		58.4%	0.006
No	10	1236	2.34[1.86–2.96]	2.34[1.86–2.96]	0.038	0.00%	0.615
Yes	2	375	0.927[0.551–1.56]	0.953[0.199–4.57]		89.0%	0.003
**Quality score (%)**							
<85.0	8	877	2.28[1.78–2.92]	2.28[1.78–2.92]	0.316	71.4%	0.001
≥85.0	8	855	1.90[1.45–2.50]	2.07[1.19–3.59]		0.00%	0.657

In order to further explore the sources of heterogeneity, we performed meta-regression by the covariates including region, type of cancer, sample size, preoperative treatment and paper quality to quantify the heterogeneity (**Table 2**). As was found in the subgroup analysis, only the factor of preoperative treatment accounted for the inter-study heterogeneity which was consistent with the result of subgroup analysis. Moreover, HR did not change significantly after the exclusion of any of the studies in the sensitivity analysis (**Figure S1A**). For meta-analysis of the association between *HOTAIR* expression and OS, Begg's test (P = 0.022) showed significant publication bias across studies; the funnel plot was slightly asymmetrical although Egger's test (*p* = 0.103) did not show significance (**Figure S2A**).

**Figure 3:**
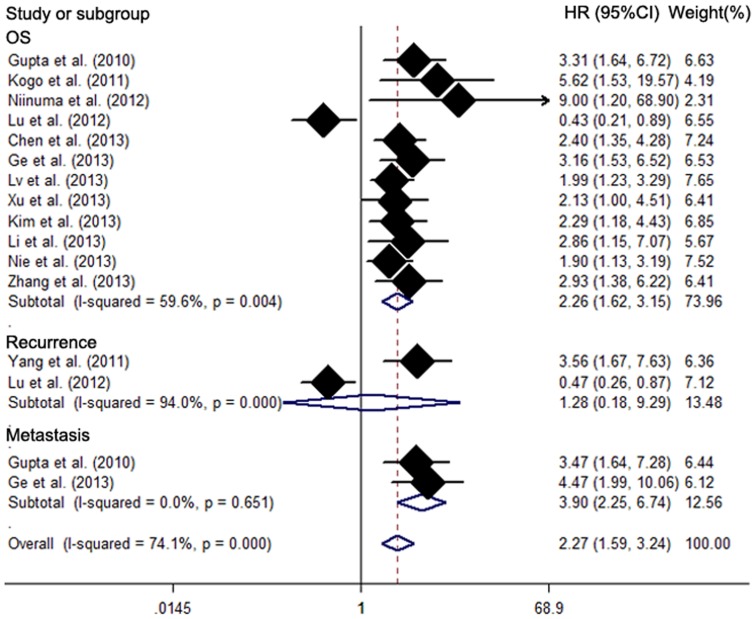
Meta analysis of the independent role of *HOTAIR* in OS/recurrence/metastasis of different types of cancer.

HRs from Cox multivariate analyses were recorded in 12 studies to investigate whether *HOTAIR* was predictive for OS of cancer. Combining these HRs suggests that *HOTAIR* expression might be an independent prognostic factor for cancer patients (pooled HR 2.26, 95%CI: 1.62–3.15) (**Figure 3**), but a significant heterogeneity was detected among studies (χ^2^ = 27.25, df = 11, *p* = 0.004, I^2^ = 59.6%). In addition to the independent role of *HOTAIR* in OS, two other studies respectively found that *HOTAIR* was an independent factor for cancer metastasis but not for the recurrence of cancer (pooled HR 3.90, 95%CI: 2.25–6.74; pooled HR 1.28, 95%CI: 0.18–9.29) (**Table 3**) (**Figure 3**). Heterogeneity was significant in studies examining the association between *HOTAIR* and recurrence, whereas no heterogeneity was found in studies looking at the independent role of *HOTAIR* in metastasis.

**Table 3:** Results of subgroup analysis of the independent role of *HOTAIR* in overall survival/recurrence/metastasis of different types of cancer.

Subgroup analysis	No. of studies	No. of patients	Pooled HR(95%CI)		Meta regression (*p*-value)	Heterogeneity	
			Fixed	Random		I^2^	*p*-value
**Overall survival**	12	1377	2.15[1.76–2.63]	2.26[1.62–3.15]	—	59.6%	0.004
**Region**							
Asian countries	9	883	2.42[1.94–3.03]	2.42[1.94–3.03]	0.185	0.00%	0.774
Western countries	3	494	1.32[0.839–2.08]	1.66[0.405–6.79]		88.2%	0.000
**Sample size**							
<100	6	454	2.38[1.79–3.17]	2.38[1.79–3.17]	0.554	0.00%	0.000
≥100	6	923	1.95[1.47–2.59]	2.08[1.10–3.93]		78.8%	0.000
**Type of cancer**							
Digestive system carcinoma	7	632	2.45[1.87–3.20]	2.45[1.87–3.20]	0.326	0.00%	0.599
Non-digestive system carcinoma	5	745	1.82[1.34–2.47]	1.85[0.905–3.78]		80.6%	0.000
**Preoperative treatment**	8	1015	1.94[1.54–2.45]	2.00[1.29–3.10]		68.8%	0.002
**No**	6	640	2.34[1.80–3.04]	2.34[1.80–3.04]	0.069	0.00%	0.591
**Yes**	2	375	0.927[0.551–1.56]	0.953[0.199–4.57]		89.0%	0.003
**Quality score (%)**							
<85.0	4	404	2.36[1.71–3.26]	2.36[1.71–3.26]	0.746	0.00%	0.734
≥85.0	8	973	2.03[1.57–2.63]	2.22[1.31–3.75]		72.5%	0.001
**Recurrence**	2	352	1.03[0.64–1.65]	1.28[0.18–9.29]	—	94.0%	0.000
**Metastasis**	2	269	3.90[2.25–6.74]	3.90[2.25–6.74]	—	0.00%	0.065

Subgroup analysis, sensitivity analysis and meta-regression were performed to illustrate the heterogeneity across studies concerning the independent role of *HOTAIR* in OS, but not in the recurrence or metastasis (**Table 3**). Subgroup analysis showed that *HOTAIR* was an independent prognostic factor for digestive system cancer patients without preoperative treatment in Asian countries, and sample size and paper quality did not change the overall result. However, we found that none of the examined factors, including region, sample size, type of cancer, preoperative treatment and manuscript quality, were responsible for heterogeneity across studies in meta-regression. Sensitivity analysis showed no significant change after exclusion any of the included studies (**Figure S1B**). There was no significant publication bias for studies concerning an independent prognostic role for *HOTAIR* in different types of cancer (Begg's test: *p* = 0.064, Egger's test: *p* = 0.25) (**Figure S2B**).

**Figure 4:**
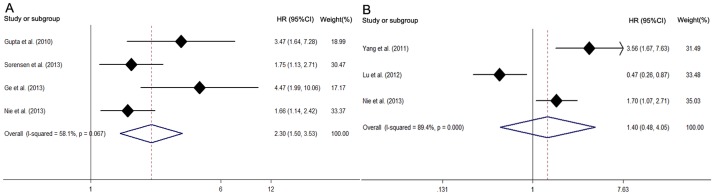
(A) Meta analysis of pooled hazard ratios of RFS of cancer with increased *HOTAIR* expression. (B) Meta analysis of pooled hazard ratios of MFS of cancer with increased *HOTAIR* expression.

The prognostic significance of *HOTAIR* in recurrence-free survival (RFS) and metastasis-free survival (MFS) was evaluated in 3 studies with 512 patients and in 4 studies with 593 patients, respectively (**Table 4**). *HOTAIR* was not significantly associated with RFS (HR 1.40, 95%CI: 0.48–4.05) with obvious heterogeneity (χ^2^ = 18.95, df = 2, *p* = 0.00, I^2^ = 89.4%)(**Figure 4A**), but a significant relation was demonstrated in the subgroup of Asian countries (HR 2.31, 95%CI: 1.13–4.71), the subgroup of sample size less than 100 (HR 3.56, 95%CI: 1.67–7.63) and the subgroup of digestive system carcinoma (HR 3.56, 95%CI: 1.67–7.63). However, it showed that patients with high *HOTAIR* expression were more likely to have significantly shorter MFS (HR 2.30, 95%CI: 1.50–3.53) with heterogeneity (**Figure 4B**). Heterogeneity existed across studies in the subgroup of Asian countries (χ^2^ = 2.64, df = 1, *p* = 0.104; I^2^ = 62.1%) while there was only one study in the Western subgroup with digestive system carcinoma. The subgroup of digestive system carcinoma (HR 4.47, 95%CI: 1.99–10.05 *vs*. HR 1.93, 95%CI: 1.36–2.74) and paper quality less than 85.0 (HR 4.47, 95%CI: 1.99–10.05 *vs*. HR 1.93, 95%CI: 1.36–2.74) reported larger HR than did the other two subgroups without significant heterogeneity. However, patients from Asian and western countries showed similar HR from *HOTAIR* in MFS with heterogeneity. Meta regression analysis showed that no included stratifying factors contributed to main heterogeneity across studies. Furthermore, we estimated HRs in 4 studies with available multivariate data regarding the independent prognostic role of *HOTAIR* in recurrence and metastasis (**Figure 3**). This analysis showed that *HOTAIR* was an independent prognostic factor for cancer metastasis (HR 3.90, 95%CI: 2.25–6.74) without heterogeneity but not for cancer recurrence (HR 1.28, 95%CI: 0.18–9.29). Sensitivity analysis changed after omitting any of the included studies in this part (**Figure S1C and 1D**). Meta-regression was not applicable in analysis of the association between *HOTAIR* and RFS because of the limited number of included studies. There was no significant publication bias across studies in analyzing *HOTAIR* and RFS (*p* = 1.00 in Begg's test and P = 0.74 in Egger's test) (**Figure S2C**). Publication bias was significant in studies regarding the association between *HOTAIR* and MFS, with a *p* value less than 0.05 in Egger's test and asymmetry of the funnel plot, although Begg's test demonstrated a *p* value larger than 0.05 (**Figure S2D**).

**Table 4:** Results of subgroup analysis of pooled hazard ratios of recurrence-free survival and metastasis-free survival of cancer with increased *HOTAIR* expression.

Subgroup analysis	No. of studies	No. of patients	Pooled HR(95%CI)		Meta regression (*p*-value)	Heterogeneity	
			Fixed	Random		I^2^	*p*-value
**Recurrence-free survival**	3	512	1.33[0.96–1.85]	1.40[0.48–4.05]	—	89.4%	0.000
**Region**							
Asian countries	2	220	2.08[1.40–3.09]	2.31[1.13–4.71]	—	62.1%	0.104
Western countries	1	292	0.47[0.26–0.86]	0.47[0.26–0.86]		—	—
**Sample size**							
<100	1	60	3.56[1.67–7.63]	3.56[1.67–7.63]	—	—	—
≥100	2	452	0.91[0.26–3.21]	1.06[0.73–1.53]		90.9%	0.001
**Type of cancer**							
Digestive system carcinoma	1	60	3.56[1.67–7.63]	3.56[1.67–7.63]	—	—	—
Non-digestive system carcinoma	2	452	0.91[0.26–3.21]	1.06[0.73–1.53]		90.9%	0.001
**Metastasis-free survival**	4	593	2.03[1.57–2.61]	2.30[1.50–3.53]	—	58.1%	0.067
**Region**							
Asian countries	2	297	1.98[1.41–2.79]	2.55[0.98–6.65]	0.831	78.7%	0.030
Western countries	2	296	2.09[1.43–3.05]	2.30[1.19–4.44]		58.6%	0.120
**Type of cancer**							
Digestive system carcinoma	1	137	4.47[1.99–10.1]	4.47[1.99–10.1]	0.777	—	—
Non-digestive system carcinoma	3	456	1.86[1.43–2.43]	1.93[1.36–2.74]		35.5%	0.212
**Quality score (%)**							
<85.0	1	137	4.47[1.99–10.1]	4.47[1.99–10.1]	0.777	—	—
≥85.0	3	456	1.86[1.43–2.43]	1.93[1.36–2.74]		35.5%	0.212

### Association between *HOTAIR* and clinicopathological characteristics of cancer

There were seven studies examining the correlation between *HOTAIR* and clinicopathological characteristics of cancer, including 3 studies regarding esophageal carcinoma, 2 studies involving hepatocellular cancer and 2 studies of gastric cancer (**Table 5**). In esophageal carcinoma, high *HOTAIR* expression was significantly associated with TNM stage (III/IV *vs*. I/II: OR 6.90, 95%CI: 2.81–16.9) and N status (N2/3 *vs*. N0/1: OR 3.29, 95% CI:1.18–9.16) whereas no significant correlation was found with T classification (T3/4 *vs*. T1/2: OR 2.15, 95% CI: 0.24–19.5) or grade of differentiation (G3/4 *vs*. G1/2: OR 1.14, 95% CI: 0.10–13.0). The analysis between *HOTAIR* expression and T classification, N status and grade of differentiation in esophageal carcinoma displayed significant heterogeneity across studies except TNM stage. However, we did not observe a significant correlation between *HOTAIR* and TNM stage (III/IV vs. I/II: OR 0.92, 95%CI: 0.40–2.12) and invasion of the portal vein (positive *vs*. negative: OR 1.23, 95%CI: 0.55–2.78) in hepatocellular cancer. This result was strengthened by the low heterogeneity between studies. In terms of gastric cancer, *HOTAIR* expression was found to be significantly associated with lymph node metastasis (present *vs*. absent: OR 4.47, 95%CI:1.88–10.63) and vessel invasion (positive vs. negative: OR 2.88, 95%CI: 1.38–6.04) while TNM stage (III/IV vs. I/II: OR 1.94, 95%CI:0.65–5.85) and depth of invasion (T3/4 vs. T1/2: OR 1.32, 95%CI:0.63–2.77) tended to have relatively weaker correlations with *HOTAIR* expression. Except for TNM stage, there was no significant heterogeneity between studies in lymph node metastasis, vessel invasion and depth of invasion. Subgroup analysis, sensitivity analysis, meta-regression analysis and assessment of publication bias was not performed due to the relatively little heterogeneity across studies and limited number of included papers.

**Table 5:** Results of meta-analysis of increased *HOTAIR* expression and clinicopathological features of three types of cancer.

Clinicopathological features	No. of studies	No. of patients	Pooled OR		*p*-value	Heterogeneity	
			Fixed	Random		I^2^	*p*-value
**Esophageal carcinoma**							
TNM stage	2	171	6.93[2.79–17.2]	6.90[2.81–16.94]	0.000	0.00%	0.953
T classification	2	230	1.60[0.791–3.27	2.15[0.237–19.5]	0.497	83.0%	0.015
N status	3	215	2.84[1.73–4.67]	3.29[1.18–9.16]	0.023	74.3%	0.020
Grade of differentiation	2	230	0.75[0.366–1.55]	1.14[0.101–13.0]	0.915	85.1%	0.010
**Hepatocellular carcinoma**							
TNM stage	2	124	0.92[0.398–2.12]	0.917[0.398–2.12]	0.840	0.00%	0.967
Invasion of portal vein	2	124	1.23[0.547–2.78]	1.23[0.545–2.78]	0.616	0.00%	0.765
**Gastric cancer**							
TNM stage	2	151	1.92[0.959–3.83]	1.94[0.646–5.85]	0.238	59.6%	0.116
Lymph node metastasis	2	151	4.40[1.99–9.71]	4.47[1.88–10.6]	0.001	13.6%	0.282
Depth of invasion	2	151	1.65[0.601–4.55]	1.32[0.625–2.77]	0.470	0.00%	0.513
Vessel invasion	2	151	1.01[0.335–3.01]	2.88[1.38–6.04]	0.005	2.10%	0.312

## Discussion

In recent years, numerous studies have demonstrated that lincRNAs are involved in various biological processes, including cancer progression and metastasis, via chromosome remodeling, transcription and post-transcriptional processing [36]. The lincRNA *HOTAIR* is aberrantly expressed in different types of cancer. In this meta-analysis, we have examined the prognostic role of *HOTAIR* in cancer and the relation between *HOTAIR* and clinicopathological characteristics of cancer. We believe that this meta-analysis is the first to investigate the relationship between a lincRNA and cancer prognosis. The fact that we included studies from both Asian and Western countries may enhance the generalizability to some extent. Subgroup analysis in a fixed or random model, meta regression analysis and sensitivity analysis were all performed in the current study, enhancing the statistical power to detect a role of *HOTAIR* in different types of cancer.

A total of 19 papers comprising 2033 patients were included into this meta-analysis. We found that *HOTAIR* expression was associated with a poorer prognosis in patients with different types of cancer. Since significant heterogeneity existed across these studies, subgroup analyses were performed. Factors including region (Asian or western countries), type of cancer (digestive or non-digestive disease), sample size (more or less than 100), and paper quality (score more or less than 85%) did not alter the significant predictive value of *HOTAIR* expression in OS in different kinds of cancer. By combining HRs from Cox multivariate analyses, we found that *HOTAIR* was an independent prognostic factor for cancer patients (pooled HR 2.26, 95% CI: 1.62–3.15). However, heterogeneity existed. Subgroup analysis showed that sample size and paper quality did not change the overall result, but that the type of cancer, preoperative treatment and region did. In addition to this, biological types of carcinoma might also be a resource of heterogeneity which was not analyzed in our study due to limited data. For example, a majority of participants in the studies on breast cancer were estrogen-receptor (ER) positive and progesterone receptor (PR) positive, and it is theoretically possible that the prognostic value of *HOTAIR* expression might be different in ER and PR-negative breast cancer. Of note, the prognostic significance of *HOTAIR* in OS was observed in patients without preoperative treatment rather than those with preoperative treatment which to some extent showed the true prognostic value of *HOTAIR* in survival with a controlled population in which a treatment modality modified the predictive effect of *HOTAIR*. *HOTAIR* could be used as an independent prognostic factor for digestive system cancer patients in Asian countries without heterogeneity. Both Begg's test and Egger's test found no significant publication bias concerning on independent prognostic role of *HOTAIR* in different types of cancer.

TNM stage is associated with cancer prognosis. In this meta-analysis, only seven studies examined the correlation between *HOTAIR* and TNM stage. Among these, 3 studies investigated esophageal carcinoma. We found that high *HOTAIR* expression in esophageal carcinoma was significantly associated with TNM stage (III/IV vs. I/II: OR 6.90, 95%CI: 2.81–16.9) without obvious heterogeneity.

Previous studies have shown that N status, vessel invasion and depth of invasion were associated with an unfavorable outcome in cancer patients [37]–[39]. In our meta-analysis, we found that in gastric cancer, *HOTAIR* expression was significantly associated with lymph node metastasis and vessel invasion without heterogeneity. However, no such association was found in liver cancer or esophageal cancer. One potential explanation for these differences might be that the number of included studies was small. Therefore, more studies should be conducted in order to clarify the relationship between *HOTAIR* and clinicopathological features in other types of cancer.

The prognostic significance of *HOTAIR* in RFS and MFS was evaluated in 3 studies with 529 patients and in 4 studies with 593 patients, respectively. Subgroup analysis showed that patients with high *HOTAIR* expression were more likely to have significantly shorter MFS albeit with heterogeneity, but *HOTAIR* expression was not significantly associated with poorer RFS. Considering the limited number of studies concerning the relationship between *HOTAIR* and RFS or MFS, we cannot draw a definite conclusion regarding the relationship, as more studies with large sample size are needed.

Since *HOTAIR* overexpression in breast cancer cells promoted cancer cell invasion, and *HOTAIR* silencing reduced cancer invasiveness through Matrigel in vitro [6], we estimated HRs in 4 studies with available multivariate data regarding the independent prognostic role of *HOTAIR* in recurrence and metastasis. We show that *HOTAIR* is an independent prognostic factor for cancer metastasis (HR 3.90, 95%CI: 2.25–6.74) without heterogeneity.

Through subgroup analysis, we identified for the first time that *HOTAIR* was a novel predictive factor for poor prognosis in different types of cancers for both Western and Asian populations; however, *HOTAIR* was an independent prognostic factor for OS of Asian patients rather than Western ones. Secondly, we found that the predictive significance of *HOTAIR* in OS, RFS and MFS was more significant in patients with digestive system carcinoma than in those with non-digestive system carcinoma. The above two findings suggest that *HOTAIR* expression might be more meaningful in predicting OS of Asian patients or patients with digestive system carcinoma than that of Western patients or those with non-digestive system cancer. Finally, we showed that the pooled HRs in the studies with poorer quality were larger than those in the studies with better quality, suggesting that the results in some individual studies with poor quality might overestimate the predictive effect of *HOTAIR*.

It should be emphasized that there are several limitations in our study. First, the cut-off value of high and low *HOTAIR* expression varied in different studies. It was difficult to reach a consensus value. Second, the treatment protocols after surgery differed in the various studies, and these differences might have a great impact on survival and thus result in some heterogeneity. Third, most of the HRs could not be directly obtained from the primary studies, requiring us to calculate them ourselves or to reconstruct the survival curves to extract the HR estimates. Fourth, we only included English language papers. Fifth, most of the included studies reported positive results because those with negative results are generally less likely to be published. Thus, our results might overestimate the predictive significance of *HOTAIR* in prognosis of cancer to some extent. Sixth, differences of paper quality across the studies might have led to bias in the meta-analysis although subgroup analysis and meta regression did not show the paper quality as the resource of heterogeneity. Seventh, we could not investigate the role of *HOTAIR* in different biological subtypes of a given cancer as this distinction was not available for most studies.

In conclusion, our study found that *HOTAIR* might be a novel predictive factor for assessing poor prognosis in different types of cancer both in Asian and western countries. This is the first example of a lincRNA being shown to be a biomarker in predicting cancer prognosis.

## Supporting Information

Figure S1 **Sensitivity analysis of the association between HOTAIR expression and OS/RFS/MFS of cancer.** (A) Sensitivity analysis of the pooled HRs of OS of different types of cancer with increased *HOTAIR* expression; (B) Sensitivity analysis of the independent role of *HOTAIR* in OS/recurrence/metastasis of different types of cancer; (C) Sensitivity analysis of the pooled HRs of RFS of cancer with increased *HOTAIR* expression; (D) Sensitivity analysis of the pooled HRs of MFS of cancer with increased *HOTAIR* expression.
(TIF)

Figure S2 **Funnel plot for the analysis of the association between HOTAIR expression and OS/RFS/MFS of cancer.** (A)Funnel plot for the analysis of the association between HOTAIR expression and OS of cancer; (B) Funnel plot for the analysis of independent prognostic role of HOTAIR in different types of cancer; (C) Funnel plot for the analysis of the association between HOTAIR expression and RFS of cancer; (D) Funnel plot for the analysis of the association between HOTAIR expression and MFS of cancer.
(TIF)

Checklist S1 **PRISMA checklist. Each section was localized in the paper.**
(DOC)

## References

[1] Globocan (2012) IARC. http://globocan.iarc.fr/Pages/fact_sheets_cancer.aspx. Accessed July 18, 2014.

[2] Bray F, Ren JS, Masuyer E, Ferlay J (2013) Global estimates of cancer prevalence for 27 sites in the adult population in 2008. Int J Cancer 132: 1133–1145. 22752881 10.1002/ijc.27711

[3] Gonzalez CA, Agudo A (2012) Carcinogenesis, prevention and early detection of gastric cancer: where we are and where we should go. Int J Cancer 130: 745–753. 21918974 10.1002/ijc.26430

[4] Guttman M, Donaghey J, Carey BW, Garber M, Grenier JK, et al (2011) lincRNAs act in the circuitry controlling pluripotency and differentiation. Nature 477: 295–300. 21874018 10.1038/nature10398 PMC3175327

[5] Rinn JL, Kertesz M, Wang JK, Squazzo SL, Xu X, et al (2007) Functional Demarcation of Active and Silent Chromatin Domains in Human HOX Loci by Noncoding RNAs. Cell 129: 1311–1323. 17604720 10.1016/j.cell.2007.05.022 PMC2084369

[6] Gupta RA, Shah N, Wang KC, Kim J, Horlings HM, et al (2010) Long non-coding RNA HOTAIR reprograms chromatin state to promote cancer metastasis. Nature 464: 1071–1076. 20393566 10.1038/nature08975 PMC3049919

[7] Kogo R, Shimamura T, Mimori K, Kawahara K, Imoto S, et al (2011) Long Noncoding RNA HOTAIR Regulates Polycomb-Dependent Chromatin Modification and Is Associated with Poor Prognosis in Colorectal Cancers. Cancer Res 71: 6320–6326. 21862635 10.1158/0008-5472.CAN-11-1021

[8] Li D, Feng J, Wu T, Wang Y, Sun Y, et al (2013) Long Intergenic Noncoding RNA HOTAIR Is Overexpressed and Regulates PTEN Methylation in Laryngeal Squamous Cell Carcinoma. Am J Pathol 182: 64–70. 23141928 10.1016/j.ajpath.2012.08.042

[9] Yang Z, Zhou L, Wu LM, Lai MC, Xie HY, et al (2011) Overexpression of Long Non-coding RNA HOTAIR Predicts Tumor Recurrence in Hepatocellular Carcinoma Patients Following Liver Transplantation. Ann Surg Oncol 18: 1243–1250. 21327457 10.1245/s10434-011-1581-y

[10] McShane LM, Altman DG, Sauerbrei W, Taube SE, Gion M, et al (2005) Statistics Subcommittee of the NCI-EORTC Working Group on Cancer Diagnostics. Reporting recommendations for tumor marker prognostic studies (REMARK). J Natl Cancer Inst 97: 1180–1184. 16106022 10.1093/jnci/dji237

[11] Altman DG, McShane LM, Sauerbrei W, Taube SE (2012) Reporting Recommendations for Tumor Marker Prognostic Studies (REMARK): explanation and elaboration. PLoS Med 9: e1001216. 22675273 10.1371/journal.pmed.1001216 PMC3362085

[12] Steels E, Paesmans M, Berghmans T, et al (2001) Role of p53 as a prognostic factor for survival in lung cancer: a systematic review of the literature with a meta-analysis. Eur Respir J 18: 705–719. 11716177 10.1183/09031936.01.00062201

[13] Xing X, Tang YB, Yuan G, Wang Y, Wang J, et al (2013) The prognostic value of E-cadherin in gastric cancer: a meta-analysis. Int J Cancer 132: 2589–2596. 23169395 10.1002/ijc.27947

[14] Tierney JF SL, Ghersi D, Burdett S, Sydes MR (2007) Practical methods for incorporating summary time-to-event data into meta-analysis. Trials 8: 16. 17555582 10.1186/1745-6215-8-16 PMC1920534

[15] Higgins JP, Thompson SG, Deeks JJ, Altman DG (2003) Measuring inconsistency in meta-analyses. BMJ 327: 557–560. 12958120 10.1136/bmj.327.7414.557 PMC192859

[16] Parmar MK, Torri V, Stewart L (1998) Extracting summary statistics to perform meta-analyses of the published literature for survival endpoints. Stat Med 17: 2815–2834. 9921604 10.1002/(sici)1097-0258(19981230)17:24<2815::aid-sim110>3.0.co;2-8

[17] Yusuf S, Peto R, Lewis J, Collins R, Sleight P (1985) Beta blockade during and after myocardial infarction: an overview of the randomized trials. Prog Cardiovasc Dis 27: 335–371. 2858114 10.1016/s0033-0620(85)80003-7

[18] Egger M, Davey Smith G, Schneider M, Minder C (1997) Bias in meta-analysis detected by a simple, graphical test. BMJ 315: 629–634. 9310563 10.1136/bmj.315.7109.629 PMC2127453

[19] Chen FJ, Sun M, Li SQ, Wu QQ, Ji L, et al (2013) Upregulation of the long non-coding RNA HOTAIR promotes esophageal squamous cell carcinoma metastasis and poor prognosis. Mol Carcinog 52: 908–915. 24151120 10.1002/mc.21944

[20] Li X, Wu Z, Mei Q, Guo M, Fu X, et al (2013) Long non-coding RNA HOTAIR, a driver of malignancy, predicts negative prognosis and exhibits oncogenic activity in oesophageal squamous cell carcinoma. Br J Cancer 109: 2266–2278. 24022190 10.1038/bjc.2013.548 PMC3798955

[21] Lv XB, Lian GY, Wang HR, Song E, Yao H, et al (2013) Long noncoding RNA HOTAIR is a prognostic marker for esophageal squamous cell carcinoma progression and survival. PLoS One 8: e63516. 23717443 10.1371/journal.pone.0063516 PMC3662674

[22] Nie Y, Liu X, Qu S, Song E, Zou H, et al (2013) Long non-coding RNA HOTAIR is an independent prognostic marker for nasopharyngeal carcinoma progression and survival. Cancer Sci 104: 458–464. 23281836 10.1111/cas.12092 PMC7657223

[23] Lu L, Zhu G, Zhang C, Deng Q, Katsaros D, et al (2012) Association of large noncoding RNA HOTAIR expression and its downstream intergenic CpG island methylation with survival in breast cancer. Breast Cancer Res Treat 136: 875–883. 23124417 10.1007/s10549-012-2314-z

[24] Kim K, Jutooru I, Chadalapaka G, Johnson G, Frank J, et al (2012) HOTAIR is a negative prognostic factor and exhibits pro-oncogenic activity in pancreatic cancer. Oncogene 32: 1616–1625. 22614017 10.1038/onc.2012.193 PMC3484248

[25] Nakagawa T, Endo H, Yokoyama M, Abe J, Tamai K, et al (2013) Large noncoding RNA HOTAIR enhances aggressive biological behavior and is associated with short disease-free survival in human non-small cell lung cancer. Biochem Biophys Res Commun 436: 319–324. 23743197 10.1016/j.bbrc.2013.05.101

[26] He X, Bao W, Li X, Chen Z, Che Q, et al (2014) The long non-coding RNA HOTAIR is upregulated in endometrial carcinoma and correlates with poor prognosis. Int J Mol Med 33: 325–332. 24285342 10.3892/ijmm.2013.1570

[27] Sørensen KP, Thomassen M, Tan Q, Bak M, Cold S, et al (2013) Long non-coding RNA HOTAIR is an independent prognostic marker of metastasis in estrogen receptor-positive primary breast cancer. Breast Cancer Res Treat 142: 529–536. 24258260 10.1007/s10549-013-2776-7

[28] Endo HST, Nakagawa T, Yokoyama M, Tamai K, Yamanami H, et al (2013) Enhanced expression of long non-coding RNA HOTAIR is associated with the development of gastriccancer. PLoS One 8: e77070. 24130837 10.1371/journal.pone.0077070 PMC3795022

[29] Liu XH, Liu ZL, Sun M, Liu J, Wang ZX, et al (2013) The long non-coding RNA HOTAIR indicates a poor prognosis and promotes metastasis in non-small cell lung cancer. BMC Cancer 13: 464. 24103700 10.1186/1471-2407-13-464 PMC3851855

[30] Niinuma T, Suzuki H, Nojima M, Nosho K, Yamamoto H, et al (2012) Upregulation of miR-196a and HOTAIR Drive Malignant Character in Gastrointestinal Stromal Tumors. Cancer Research 72: 1126–1136. 22258453 10.1158/0008-5472.CAN-11-1803

[31] Ge XS, Ma HJ, Zheng XH, Ruan HL, Liao XY, et al (2013) HOTAIR, a prognostic factor in esophageal squamous cell carcinoma, inhibits WIF-1 expression and activates Wnt pathway. Cancer Sci 104: 1675–1682. 24118380 10.1111/cas.12296 PMC7653522

[32] Zhang JX, Han L, Bao ZS, Wang YY, Chen LY, et al (2013) HOTAIR, a cell cycle-associated long noncoding RNA and a strong predictor of survival, is preferentially expressed in classical and mesenchymal glioma. Neuro Oncol 15: 1595–1603. 24203894 10.1093/neuonc/not131 PMC3829598

[33] Xu ZY, Yu QM, Du YA, Yang LT, Dong RZ, et al (2013) Knockdown of Long Non-coding RNA HOTAIR Suppresses Tumor Invasion and Reverses Epithelial-mesenchymal Transition in Gastric Cancer. Int J Biol Sci 9: 587–597. 23847441 10.7150/ijbs.6339 PMC3708039

[34] Ishibashi M, Kogo R, Shibata K, Sawada G, Takahashi Y, et al (2013) Clinical significance of the expression of long non-coding RNA HOTAIR in primary hepatocellular carcinoma. Oncol Rep 29: 946–950. 23292722 10.3892/or.2012.2219

[35] Geng YJ, Xie SL, Li Q, Ma J, Wang GY (2011) Large Intervening Non-Coding RNA HOTAIR is Associated with Hepatocellular Carcinoma Progression. J Int Med Res 39: 2119–2128. 22289527 10.1177/147323001103900608

[36] Shi X, Sun M, Liu H, Yao Y, Song Y (2013) Long non-coding RNAs: a new frontier in the study of human diseases. Cancer Lett 339: 159–166. 23791884 10.1016/j.canlet.2013.06.013

[37] Laerum OD, Ovrebo K, Skarstein A, Christensen IJ, Alpizar-Alpizar W, et al (2012) Prognosis in adenocarcinomas of lower oesophagus, gastro-oesophageal junction and cardia evaluated by uPAR-immunohistochemistry. Int J Cancer 131: 558–569. 21866548 10.1002/ijc.26382

[38] Xu J, Zhang C, He Y, Wu H, Wang Z, et al (2012) Lymphatic endothelial cell-secreted CXCL1 stimulates lymphangiogenesis and metastasis of gastric cancer. Int J Cancer 130: 787–797. 21387301 10.1002/ijc.26035

[39] Zhang CH, Xu GL, Jia WD, Ge YS, Li JS, et al (2012) Prognostic significance of osteopontin in hepatocellular carcinoma: a meta-analysis. Int J Cancer 130: 2685–2692. 21780114 10.1002/ijc.26301

