# Supplementary figures and images for: Long Noncoding RNA HOTAIR as an Independent Prognostic Marker in Cancer: A Meta-Analysis
Source: PLoS One. 2014 Aug 26;9(8):e105538. doi: 10.1371/journal.pone.0105538 (PMC4144893; doi:10.1371/journal.pone.0105538)

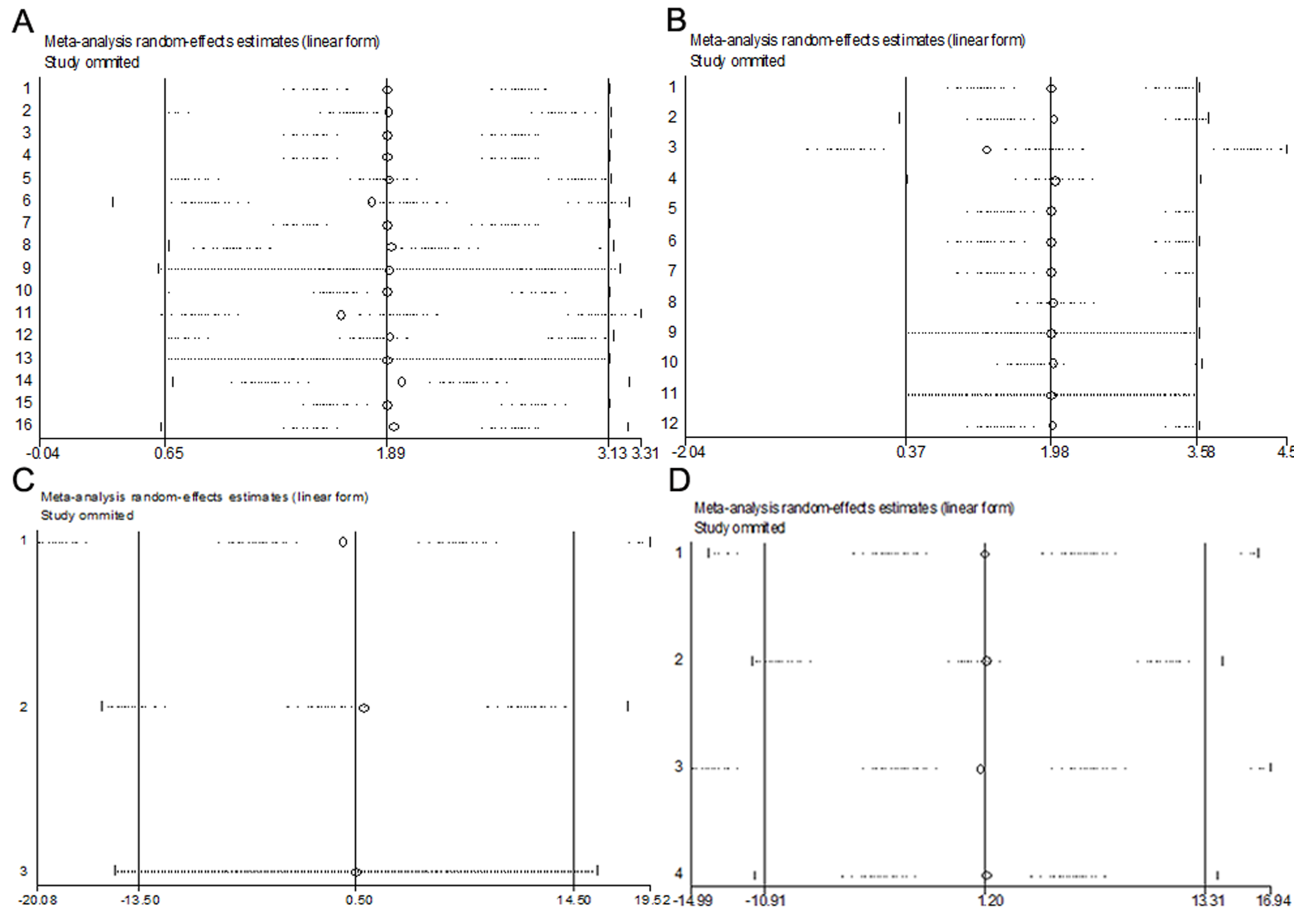

Supplement: Figure S1 — Sensitivity analysis of the association between HOTAIR expression and OS/RFS/MFS of cancer. (A) Sensitivity analysis of the pooled HRs of OS of different types of cancer with increased HOTAIR expression; (B) Sensitivity analysis of the independent role of HOTAIR in OS/recurrence/metastasis of different types of cancer; (C) Sensitivity analysis of the pooled HRs of RFS of cancer with increased HOTAIR expression; (D) Sensitivity analysis of the pooled HRs of MFS of cancer with increased HOTAIR expression. (TIF) [file pone.0105538.s001.tif]

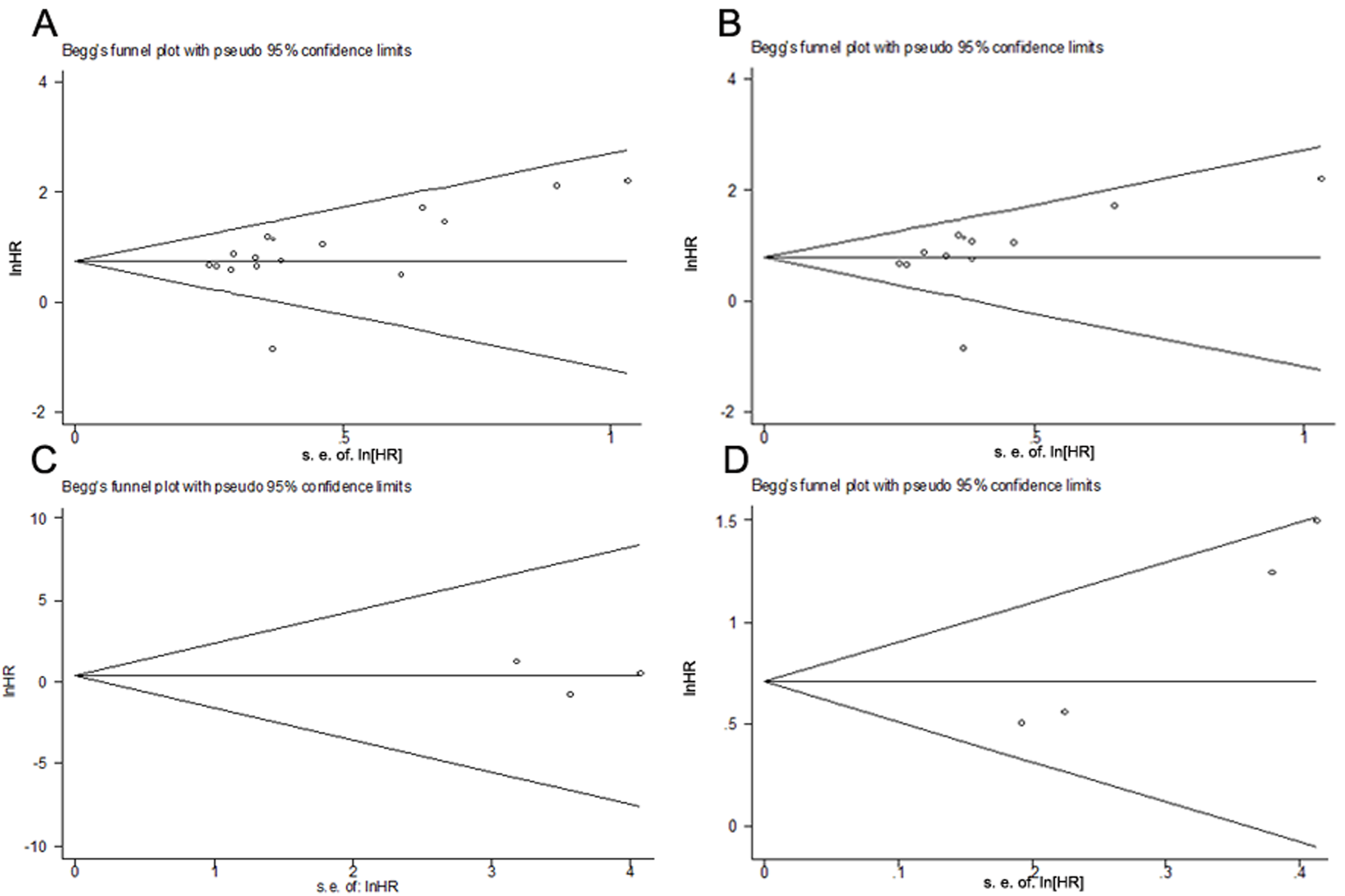

Supplement: Figure S2 — Funnel plot for the analysis of the association between HOTAIR expression and OS/RFS/MFS of cancer. (A)Funnel plot for the analysis of the association between HOTAIR expression and OS of cancer; (B) Funnel plot for the analysis of independent prognostic role of HOTAIR in different types of cancer; (C) Funnel plot for the analysis of the association between HOTAIR expression and RFS of cancer; (D) Funnel plot for the analysis of the association between HOTAIR expression and MFS of cancer. (TIF) [file pone.0105538.s002.tif]
